# Supplementary material for: Novel Driver Strength Index highlights important cancer genes in TCGA PanCanAtlas patients
Source: PeerJ. 2022 Aug 11;10:e13860. doi: 10.7717/peerj.13860 (PMC9375969; doi:10.7717/peerj.13860)
Supplement: Supplemental Information 2 [file peerj-10-13860-s002.zip › COHORTS/genes plots/cohorts/2021_8_16_14_9_distribution_cohorts_males_KIRC.pdf]

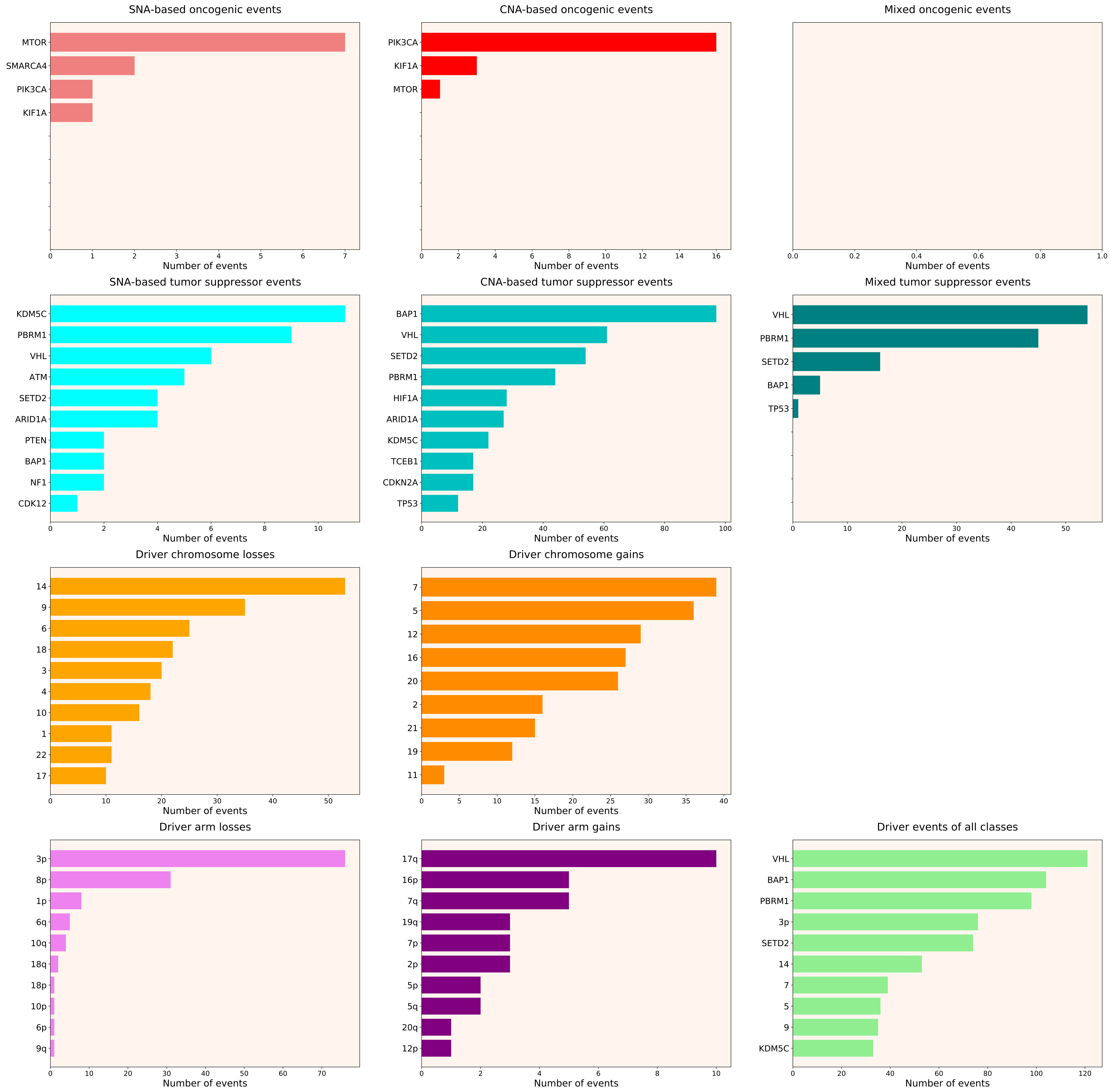

SNA-based oncogenic events
MTOR
SMARCA4
PIK3CA
KIF1A
Number of events
CNA-based oncogenic events
PIK3CA
KIF1A
MTOR
Number of events
Mixed oncogenic events
Number of events
SNA-based tumor suppressor events
KDM5C
PBRM1
VHL
ATM
SETD2
ARID1A
PTEN
BAP1
NF1
CDK12
Number of events
CNA-based tumor suppressor events
BAP1
VHL
SETD2
PBRM1
HIF1A
ARID1A
KDM5C
TCEB1
CDKN2A
TP53
Number of events
Mixed tumor suppressor events
VHL
PBRM1
SETD2
BAP1
TP53
Number of events
Driver chromosome losses
Number of events
Driver chromosome gains
Number of events
Driver arm losses
3p
8p
1p
6q
10q
18q
18p
10p
6p
9q
Number of events
Driver arm gains
17q
16p
7q
19q
7p
2p
5p
5q
20q
12p
Number of events
Driver events of all classes
VHL
BAP1
PBRM1
3p
SETD2
14
7
5
9
KDM5C
Number of events
